# Co-Infection Burden of Hepatitis C Virus and Human Immunodeficiency Virus among Injecting Heroin Users at the Kenyan Coast

**DOI:** 10.1371/journal.pone.0132287

**Published:** 2015-07-24

**Authors:** Ruth S. Mwatelah, Raphael M. Lwembe, Saida Osman, Bernhards R. Ogutu, Rashid Aman, Rose C. Kitawi, Laura N. Wangai, Florence A. Oloo, Gilbert O. Kokwaro, Washingtone Ochieng

**Affiliations:** 1 Centre for Research in Therapeutic Sciences, Strathmore University, Nairobi, Kenya; 2 Institute of Tropical Medicine and Infectious Diseases, Nairobi, Kenya; 3 Jomo Kenyatta University of Agriculture and Technology, Nairobi, Kenya; 4 Centre for Virus Research, Kenya Medical Research Institute, Nairobi, Kenya; 5 Centre for Clinical Research, Kenya Medical Research Institute, Nairobi, Kenya; 6 Institute of Healthcare Management, Strathmore University, Nairobi, Kenya; 7 African Center for Clinical Trials, Nairobi, Kenya; 8 Technical University of Kenya, Nairobi, Kenya; University of Missouri-Kansas City, UNITED STATES

## Abstract

**Background:**

Injection drug use is steadily rising in Kenya. We assessed the prevalence of both human immunodeficiency virus type 1 (HIV-1) and hepatitis C virus (HCV) infections among injecting heroin users (IHUs) at the Kenyan Coast.

**Methods:**

A total of 186 IHUs (mean age, 33 years) from the Omari rehabilitation center program in Malindi were consented and screened for HIV-1 and HCV by serology and PCR and their CD4 T-cells enumerated by FACS.

**Results:**

Prevalence of HIV-1 was 87.5%, that of HCV was 16.4%, co-infection was 17.9% and 18/152 (11.8%) were uninfected. Only 5.26% of the HIV-1 negative injectors were HCV positive. Co-infection was higher among injectors aged 30 to 40 years (20.7%) and among males (22.1%) than comparable groups. About 35% of the injectors were receiving antiretroviral treatment (ART). Co-infection was highest among injectors receiving D4T (75%) compared to those receiving AZT (21.6%) or TDF (10.5%) or those not on ART (10.5%). Mean CD4 T-cells were 404 (95% CI, 365 - 443) cells/mm^3^ overall, significantly lower for co-infected (mean, 146; 95% CI 114 – 179 cells/mm^3^) than HIV mono infected (mean, 437, 95% CI 386 – 487 cells/mm^3^, p<0.001) or uninfected (mean, 618, 95% CI 549 – 687 cells/mm^3^, p<0.001) injectors and lower for HIV mono-infected than uninfected injectors (p=0.002). By treatment arm, CD4 T-cells were lower for injectors receiving D4T (mean, 78; 95% CI, 0.4 – 156 cells/mm^3^) than TDF (mean 607, 95% CI, 196 – 1018 cells/mm^3^, p=0.005) or AZT (mean 474, 95% CI -377 – 571 cells/mm^3^, p=0.004).

**Conclusion:**

Mono and dual infections with HIV-1 and HCV is high among IHUs in Malindi, but ART coverage is low. The co-infected IHUs have elevated risk of immunodeficiency due to significantly depressed CD4 T-cell numbers. Coinfection screening, treatment-as-prevention for both HIV and HCV and harm reduction should be scaled up to alleviate infection burden.

## Introduction

The risks and incidents of HIV infection remain high among certain demographics globally, including injection drug users (IDUs), commercial sex workers (CSW) and men who have sex with other men (MSM) [1–4]. Sub-Saharan Africa is home to highest proportions of these infections, but data among substance users in these settings is limited and incomprehensive [5, 6]. Eastern Africa leads in opiate use in Africa, with Kenya among the top two heroin using countries according to the 2011 World Drug Report [7]. Conservative statistics suggest that substance use continues to rise in Kenya particularly in the cosmopolitan coastal districts including Malindi, mostly influenced by tourism boom, illicit drug trafficking and escalated by cultural attributes [8–10]. In a recent national survey of about 11,000 people, 0.1% of people surveyed injected drugs, with a HIV prevalence of 6.3% [11], at least 2% higher than the general population [12]. Majority of the substance users inject heroin [9], but the numbers are often biased by underreporting [13]. Non-disclosure and stigma have also been partly responsible for the slow uptake of preventive measures to control infection among IDUs [14, 15]. Up to 50% of all Kenyan injectors share equipment and engage in multiple unprotected sex [16], behaviors that increase risk of infection and virus transmission [17]. The incidents of HIV among IDUs was estimated at an average of 3.8% in 2009, with Nairobi and Coastal regions accounting for majority of the epidemic at 5.8 and 6.1% respectively [18]. Other studies suggested that controlling HIV infection and transmission among IDUs could reduce HIV prevalence nationally by up to 30% over a 5-year period ending 2015 [19]. However, 4 years into those projections, the National AIDS and STIs Control Program showed HIV prevalence among IDUs in Nairobi and Mombasa to remain alarmingly high at 18%, which represented a 300% rise in prevalence from the earlier estimates and standing at about 3-times the national HIV prevalence [20]. Another independent review of literature showed that up to 36% of all IDUs in Kenya were positive [21]. These statistics, though discordant, point to an underlying increase in the incidents and risk of infections among the local IDU population [22–24]. HIV-1 infection in the United States and Europe is highest among same sex partners and in IDUs [25], and in countries like Brazil, higher risk for HCV infection was associated with injecting or smoking drug use [26]. There is also evidence that the prevalence of HCV might be a useful indicator for the risk of HIV infection of IDUs [27].

With an estimated 15% of the global HIV-1 infection occurring in people also living with chronic HCV infection, and poor treatment coverage among Kenyan IDUs [28, 29], we speculated that infection burden with both viruses could be much higher among this high risk population. This study aimed to understand the burden of HIV and HCV infections among heroin injectors undergoing voluntary rehabilitation care at the Omari facility in Malindi, Kenya. We show that co-infection was high and CD4 T-cells depressed among infected injectors.

## Materials and Methods

### Study site and subjects

This study was conducted at the Omari rehabilitation center (ORC) in Malindi, which is located at the Kenyan Coast in Kilifi County. This County of over 1.1 million inhabitants borders Mombasa and Kwale to the south and is dominated by the Swahili and Mijikenda tribes, but also has significant presence of Arabs, Indian and European settlers. Other major Kenyan communities are also represented substantially in the County population. Tourism is a key economic activity at the Kenyan Coast, with Malindi being one of the top destinations. ORC enrolls subjects from the general coastal communities as well as individuals actively or previously under lawful incarceration. Subjects are identified and enrolled voluntarily to the program through peer counselors, who are current or previous IDUs. The Omari Project has 3 components; (i) residential, (ii) outpatient, and (iii) outreach services covering the entire coastal region. All components offer drug and HIV/AIDS testing and education including prevention methods, risk awareness and withdrawal management. National guidelines are applicable for the management of HIV, HCV and other infections [20, 30]. The residential program provides comprehensive clinical care (CCC) management, socio-supportive care including religious and professional counseling and peer-group enrichment. The outreach component also facilitates transport to CCC for clinical management and monitoring, home detoxification, individualized counseling and Narcotics anonymous programs for lawfully free or confined users. The project offers regular and relevant training, and retains staff either internally or through partnerships with regional partner facilities. Enrollment in any of the programs is open to any voluntary participants resident within the coastal region. Thus, the study subjects represented a fair mix of local and national demographics as would be found in major cosmopolitan cities in the country. Substance use status disclosure and individual consent were the main inclusion/exclusion criteria. Other than infection, treatment data and CD4 T-cell data, no other clinical data on the IHUs was available from facility to the investigators. Secondary to disclosure extracted from facility records and for purposes of this study, structured questionnaires on substance use were administered to all participants following written informed consent. All participants were determined to be exclusive active or former heroin users undergoing withdrawal management.

### Sample collection and preparation

All consenting subjects were requested to donate a maximum of 5 milliliters of venous blood into EDTA vacutainer tubes. The blood was fractionated to separate plasma and frozen until needed. HCV screening was done twice using freshly drawn blood plasma at the field-site and repeated using frozen plasma during secondary processing at the Kenya Medical Research Institute laboratory in Nairobi. In brief, 2μL of the plasma was used for both HIV-1 and HCV detection using vironostika (biomerieux, South Africa) and diaspot (Bresta Perkasa, Indonesia) rapid serology kits respectively. The HIV-1 kits rely on the detection of anti-HIV-1 antibodies in plasma, using immobilized HIV-1 gp120, gp41 and HIV-2gp-36 antigens. The HCV kit consists of recombinant HCV antigen-coated strips that react with anti-HCV antibodies in the test plasma.

### Enumeration of CD4 T-cells

Specimens that were HIV-1 positive by these tests were subsequently enumerated for CD4 T-cell counts and the rest processed and stored for downstream applications. Enumerations were with the Alere Pima CD4 Analyser (Alere, Waltham, MA).

### Confirmation of HIV-1 infection by PCR

All HIV-1 antibody positive samples were confirmed by PCR. Plasma RNA was extracted using Qiagen viral RNA mini kit and reverse transcribed with respective HIV-1 primers using Qiagen One-step RT PCR kit. The PCR targeted the 546 base-pair envelope C2V3 region and was conducted exactly as we have reported elsewhere for non-injecting patients [31].

### Confirmation of HCV by PCR

The HCV PCR targeted a 365 base pair NS5B region of the virus and was done similarly as we reported recently [32], but for the following exceptions. For the One-step protocol, forward and reverse primer pairs used were NS5BF1-TGATACCCGCTGYTTTGACTC, (positions 7999–8020) and NS5BR1-ACCTGGTCATAGCCTCCGTGA (positions 8805–8825), with an annealing temperature of 58°C for 30 seconds and 45 PCR cycles. The inner primer pairs were NS5BF2-ATACCCGCTGYTTTGACTCAN (positions 8159–8181) and NS5BR2- GTACCTCATAGCCTCCGTG (positions 8611–8630) with annealing at 55°C for 30 seconds and 45 PCR cycles.

### Data analysis

All data was appropriately coded and entered into the SPSS platform version 20 for analysis. Age, sex, ART regimen, HIV status and HCV status were used as independent variables. The ART regimen group was divided into AZT, D4T, TDF and non-ART arms (or levels). The Infection categories were HIV/HCV dual infected, HIV mono infected, HCV mono infected and uninfected groups. The age groups were < = 30 years, 31–40 years and > 40 years. CD4 counts were used as outcome variables, and were compared between levels of independent variable. Descriptive statistics were generated using cross-tabulations and association done with Chi square test. Univariate analysis of variance (UNINOVA) and post-hoc tests were used to compare mean differences in between multiple levels age group, infection status and regimen. For infection status, HCV mono-infected sub-category (level) was excluded due to insufficient number of subjects and gender was not fitted into the model because there were less than 3 levels within the group to fit in the model. Significance is reported at p-values less than 0.05.

### Ethical approvals and consent

This study was approved by the Scientific and Ethical Review Unit C of the Kenya Medical Research Institute under two cooperate protocols, ERC/SSC #2477 and ERC/SSC #101264. Participants were adequately informed and their voluntary consent obtained in writing. Patient data were treated with strict confidence, including code labeling of questionnaires and specimen.

## Results

### Antiretroviral treatment, Substance use and clinical characteristics

A total of 186 (median and mean age 32 and 33 years respectively) injecting heroin users (IHUs) were recruited from the Omari rehabilitation Centre (Table 1). All these subjects were exclusive heroin users by self-admission. Majority of the 186 IHUs were aged below 40 years (83.3%), with more males (62.4%) than females (37.6%). About 35% of the IHUs were actively enrolled in antiretroviral treatment (ART) program. Another 65% were either not on ART or their ART status not declared in medical records. Forty-three of the 186 IHUs (23.1%) were either on maintenance cotrimoxazole (Septrin) or on cotrimoxazole preventive (prophylaxis) therapy (CPT). IHUs receiving cotrimoxazole had been diagnosed with *Pneumocystis jirovecii*, also known as *Pneumocystis carinii* (PCP) and were not on ART at the time of the report. Two other IHUs were taking the anti-malarial medication lumefantrin, plus single dose nevirapine or efavirenz. These HIV positive subjects were grouped under suboptimal ART arm for alongside those defaulting ART, and none was determined to be HCV positive. ART use within gender was comparable for both male (n = 41/116, 35.3%) and female (n = 26/70, 37.1%) injectors. ART subjects received various combinations of zidovudine (AZT), tenofovir (TDF), stavudine (D4T), lamivudine (3TC), nevirapine (NVP) and efavirenz (EFV). None of the injectors were reported to be on opioid substitution therapy and none was on HCV treatment as HCV screening was not part of routine tests conducted at local facilities.

**Table 1:** Baseline demographic and infection characteristics of subjects.

	Number, N; %N			Number, N; %N		
	*HCV+*	*HCV-*	*N*, *%T*	*HIV+*	*HIV-*	*N*, *%T*
**Age**						
< = 30 years	9; 15	51; 85	60; 39.5	64; 84.2	12; 15.8	76; 40.9
31–40 years	13; 19.4	54; 80.6	67; 44.1	68; 86.1	11; 13.9	79; 42.5
>40 years	3; 12	22; 88	25; 16.4	27; 87.1	4; 12.9	31; 16.7
**Treatment arm**						
AZT+3TC+NVP/EFV	11; 21.6	40; 78.4	51; 33.6	52; 100	0; 0	52; 28
D4T+3TC+NVP/EFV	3; 75	1; 25	4; 2.6	7; 100	0; 0	7; 3.8
TDF+3TC+NVP/EFV	0; 0	4; 100	4; 2.6	6; 100	0; 0	6; 3.2
Cotrimoxazole	4; 15.4	22; 84.6	26; 17.1	44; 100	0; 0	44; 23.7
No ART	1; 5.3	18; 94.7	19; 12.5	0; 0	27; 100	27; 14.5
ART unknown	6; 14.3	36; 85.7	42; 27.6	43; 100	0; 0	43; 23.1
Sub-optimal ART	0; 0	6; 100	6; 3.9	7; 100	0; 0	7; 3.8
**Gender**						
Male	18; 18.9	77; 81.1	95; 62.5	90; 77.6	26; 22.4	116; 62.4
Female	7; 12.3	50; 87.7	57; 37.5	69; 98.6	1; 1.4	70; 37.6
Total, T	25; 16.4	127; 83.6	152; 100	159; 85.5	27; 14.5	186; 100

Antiretroviral treatment arms: AZT, Zidovudine; D4T, Stavudine; TDF, Tenofovir; 3TC, Lamivudine; NVP, nevirapine; EFV, efavirenz. IHUs in the sub-optimal ART arm were actively taking one or both NVP or EFV instead of the standard 3-drug ART regimen.

### Co-prevalence of HIV-1 and HCV among heroin injectors

Of the 186 IHUs, 159 (85.5%) were positive for HIV by serology (Table 1). HCV screening was completed for 152 of the total 186 IHUs. Of the subjects screened for HCV, 133/152 (87.5%) were HIV positive, 25 (16.4%) were positive for HCV and 18 (11.8%) were negative for both HIV and HCV and were considered uninfected (Table 2). Of the 25 HCV infections, 24 occurred among the 133 HIV infected injectors, representing 18% co-prevalence of HCV within the HIV positive sample population. Only one of the 25 HCV positive subjects was negative for HIV. Respectively by age, HCV and HIV prevalence were 15% (9/60) and 88.3% for those aged < = 30 years, 19.4% (n = 13/67) and 86.6% for those aged 31–40 years and 12% (n = 3/25) and 88% for those aged >40 years. By genders, 18/95 (18.9%) and 77/95 (81.1%) males and 7/57 (12.3%) and 98.2% females were HCV and HIV positive respectively. Nine of 53 HIV positive injectors aged 30 years and below were also positive for HCV, constituting 17% prevalence of dual infection in this age group. Dual infection was 20.7% for injectors aged 31–40 years, 13.6% for injectors above 40 years old, 22.1% among males and 14.3% among females. Assessed differently, HIV infection was 96% among the 25 HCV positive injectors and 85.8% (109/127) for HCV negative injectors. When the 152 injectors screened for HCV were grouped by ART regimen arm, 11 of 51 (21.6%) receiving AZT, 3 of 4 (75%) receiving D4T, none (0/4) receiving TDF and 10/93 (10.5%) with no or unknown ART statuses were co-infected by both HIV and HCV. Taken together, majority of IHUs in the Omari cohort were HIV positive. The prevalence of HCV infection was comparable across all age groups, but slightly high among those older than 30 years who were also HIV positive and higher among male than female injectors. A Chi square analysis was modeled to assess associations between infection status and either age or gender. Using this approach, the proportion of males that were HIV/HCV dual infected, HIV mono-infected and uninfected was 18.1%, 63.8% and 18.1% respectively. That of female IHUs was 12.3%, 86% and 1.8% respectively. Pearson’s χ^2^ revealed a significant association between infection status and gender (p = 0.004), suggesting that the IHUs have an increased likelihood (risk) of being infected by either viruses than not being infected. This analysis yielded no significant association comparing infection status with age group (data not shown). A χ^2^ test comparing treatment arms with infection status was not meaningful since IHUs in the ‘No ART’ arm were also all ‘Not infected’.

**Table 2:** Prevalence of HIV-1 and HCV mono and dual infections among heroin injectors.

		HIV-1 status: N, %		
		Positive (+)	Negative (-)	Total, %
Category	HCV status			
< = 30 years	+	9, 17	0, 0	9, 15
	-	44, 83	7, 100	51, 85
31–40 years	+	12, 20.7	1, 11.1	13, 19.4
	-	46, 79.3	8, 88.9	54, 80.6
>40 years	+	3, 13.6	0, 0	3, 12
	-	19, 86.4	3, 100	22, 88
Mean age, years		33.17	32.95	33.14
Males	+	17, 22.1	1, 5.6	18, 18.9
	-	60, 77.9	17, 94.4	77, 81.1
Females	+	7, 12.5	0, 0	7, 12.3
	-	49, 87.5	1, 100	50, 87.3
Receiving ART*	+	14, 23.3	0, 0	14, 23.3
	-	45, 76.3	0, 0	45, 76.3
Cotrimoxazole	+	4, 15.4	0, 0	4, 15.4
	-	22, 84.6	0, 0	22, 84.6
No ART	+	0, 0	1, 5.3	1, 5.3
	-	0, 0	18, 94.7	18, 94.7
ART unknown	+	6, 14.3	0, 0	6, 14.3
	-	36, 85.7	0, 0	36, 85.7
Sub-optimal ART	+	0, 0	0, 0	0, 0
	-	6, 100	0, 0	6, 100
Overall	+	24, 18	1, 5.3	25, 16.4
	-	109, 82	18, 94.7	127, 83.5
	Total, N, %	133, 100	19, 100	152, 100
Mean age, years		33.17	32.95	33.14

*ART recipients had initiated first-line AZT, D4T or TDF triple regimen arms that included 3TC and either NVP or EFV.

### CD4 T-cell levels among heroin injectors

CD4 T-cell measurements were conducted in 172 of the total 186 IHUs, with mean counts of 404 (95% CI, 364.5–442.5) cells/mm^3^. Of the 172 injectors, 152 were infected with either or both HIV and HCV. One of the 152 was mono-infected infected with HCV, with CD4 counts of 287 cells/mm^3^. This subject did not qualify in our statistical analysis model when infection status was a variable. Table 3 shows the CD4 T-cell counts of injectors who were screened for HCV and were dual, HIV-mono or not infected. Specifically, mean CD4 counts for the D4T regimen arm was 78 (95% CI, 0.4–156) cells/mm^3^ compared to 607 (95% CI, 196–1018, p = 0.005) cells/mm^3^ for the TDF arm or 474 (95% CI, 377–571, p = 0.004) cells/mm^3^ for the AZT regimen arm. All injectors in the no-ART arm were uninfected, which explains the high CD4 counts in this category.

**Table 3:** CD4 T-cell counts compared by various categories of heroin injectors who were screened for both HIV and HCV.

	Infection Status:- Mean counts; number N, 95% CI of mean			
	HIV/HCV dual	HIV mono	Not infected	Total
**Age in yr**				
< = 30	100; 9, 48–153	479; 44, 387–570	622; 7, 542–703	439; 60, 361–516
31–40	172; 12, 127–216	396; 46, 327–465	572; 8, 426–718	376; 66, 319–434
>40	181; 3, -23–386	439; 19, 319–559	732; 3, 560–904	443; 25, 337–550
**Gender**				
Males	140; 17, 104–176	454; 60, 374–534	619; 17, 545–692	427; 94, 366–487
Females	160; 7, 72–249	416; 49, 359–473	610; 1^b^	388; 57, 333–443
**ART arm**				
AZT	205; 11, 166–243	548; 40, 435–661	0^a^ ^,^ ^b^	474; 51, 377–570
D4T	54; 3, 28–80	150; 1 ^b^	0^a^ ^,^ ^b^	78; 4, 0.4–156
TDF	0^a^	607; 4, 196–1018	0^a^ ^,^ ^b^	607; 4, 196–1018
Cotrimoxazole	156; 4, 102–209	367; 22, 293–441	0^a^ ^,^ ^b^	334; 26, 265–404
No ART	0^a^ ^,^ ^b^	0^a^ ^,^ ^b^	618; 18, 549–687	618; 18, 549–687
ART_unknown_	79; 6, 34–123	369; 36, 323–415	0^a^ ^,^ ^b^	328; 42, 277–379
_suboptimal_ART	0^a^ ^,^ ^b^	293; 6, 74–512	0^a^ ^,^ ^b^	293; 6, 74–512
Total	146; 24, 114–179	437; 109, 386–487	618; 18, 549–687	412; 151, 370–455
*p*-value†				<0.001

Legend of table:

^†^ P-value is significant at level shown comparing mean CD4 between infection statuses or between treatment arms.

^a^ No qualifying subjects.

^b^ Confidence Interval (CI) is not applicable. ART, antiretroviral treatment. Only one subject (aged 31-40yrs, CD4 T-cells of 287 counts/mm^3^) was HCV mono-infected, and is excluded from this table. Sub-optimal ART cases are IHUs with unexplained use of single-drug ART regimen.

**Fig 1:**
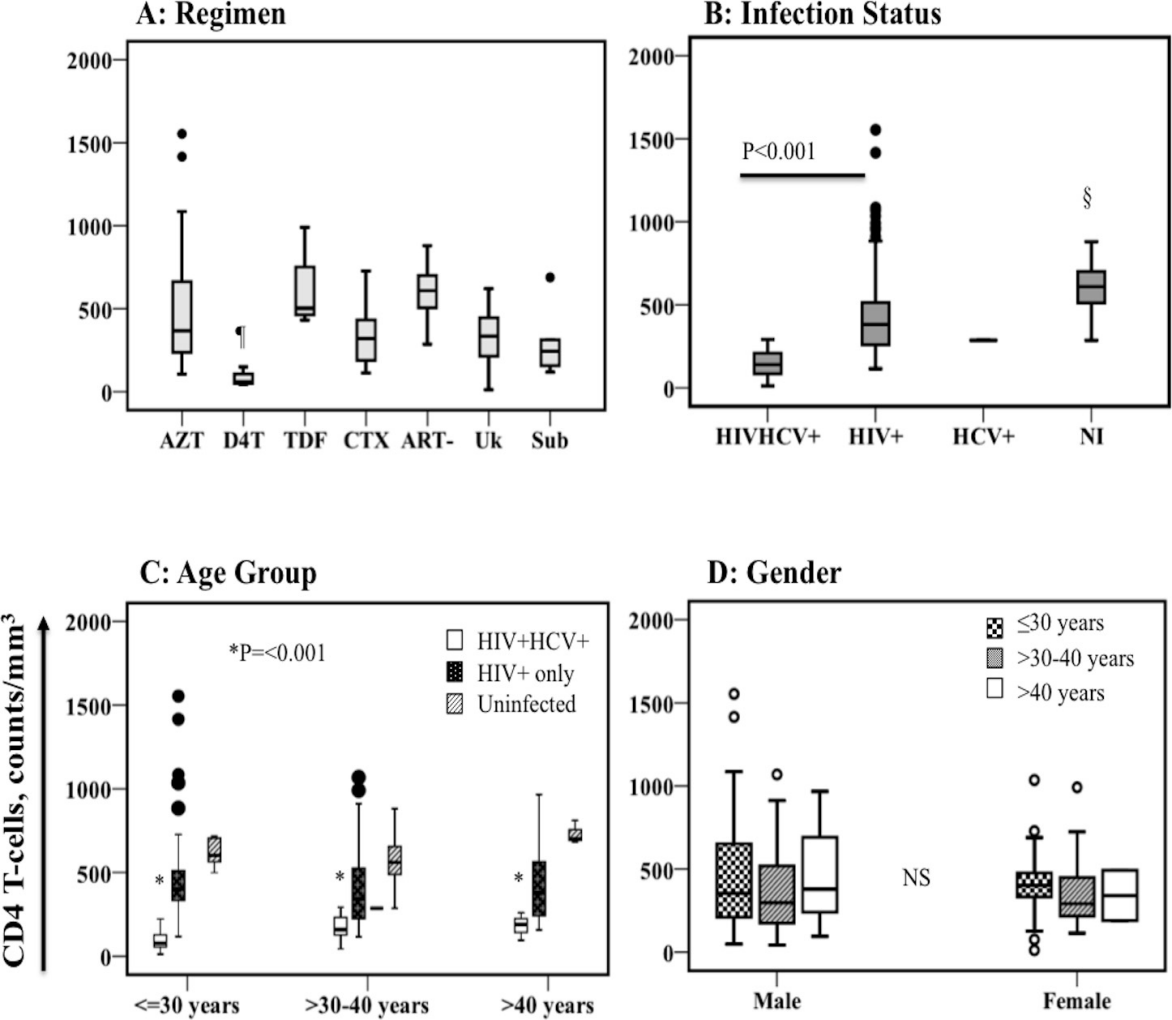
CD4 Counts across various categories of heroin injectors. CD4 T-cells are compared between treatment arms of injectors (A). ¶ Counts are significantly lower for the D4T- arm than for the AZT arm (p = 0.004), TDF (p = 0.005) or the ART- (p<0.001) arm and lower for the sub-optimal ART than ART- (p = 0.023) arm. All subjects in the ‘ART-’ arm were not infected (NI) by either virus. Mean CD4 counts are compared between infection statuses (B). These are significantly lower for co-infected than HIV-1 mono infected injectors as shown. §Shows significantly lower CD4 counts for HIV mono-infected than NI injectors (p = 0.002). CD4 data is compared between age groups of the different infection statuses (C). No significant (NS) difference was observed. *Co-infected injectors had much lower CD4 levels compared to other infection categories in any age group. Only one injector was HCV mono-infected (horizontal bar in the >30–40 years category). CD4 Counts were not significantly different between genders of injectors (D). ART, antiretroviral treatment; CTX, cotrimoxazole (septrin); ART-, No ART; Uk, unknown ART status; Sub, sub optimal ART.

By an analysis of variance (ANOVA) method, CD4 T-cells differed significantly between the ART regimen arms (p = 0.006) and infection statuses (p<0.001) but not between age groups or gender. Specific comparisons were done using post-hoc test within a Univariate ANOVA model to compare differences between multiple levels of the independent variables. By this approach, IHUs receiving D4T as part of 3-drug ART regimen had significantly depressed CD4 T-cell levels than IHUs in the other treatment arms (Fig 1A). Injectors who were dual infected with both viruses had significantly less CD4 T-cell counts (mean 146, 95% CI, 114–179 cells/mm^3^) than HIV mono-infected (mean 437, 95% CI 386–487 cells/mm^3^, p<0.001) or uninfected (mean 618, 95% CI 549–687 cells/mm^3^, p<0.001, Table 3) injectors. The difference was also significant comparing CD4 T-cells of HIV mono-infected and uninfected injectors (p = 0.002) (Fig 1B). There were no significant differences among age groups (Fig 1C) or genders (Fig 1D), although dual-infected injectors still maintained significantly low CD4 counts in any category.

## Discussion

Injection drug use (IDU) is associated with many adverse health outcomes; particularly infections with HIV-1 and HCV that continue to weigh heavily in many developing nations. In the present study, nearly twice as many males attending the Malindi rehabilitation program injected heroin as females, with all injectors using heroin. HIV-1 and HCV mono infection rates were as high as 88% and 16.4% respectively, with dual infection rate of 18%. More than twice the number of male injectors was infected with HCV as compared to their female counterparts, with CD4 T-cell counts of co-infected injectors being significantly lower than mono-infected injectors.

IDU is still a largely covert practice in Kenya, but it is increasingly recognized as one of the major drivers of the HIV epidemics in the country [17, 21]. Majority of Kenyan IDUs use heroin, which is the most widely available and accessible substance locally [9]. A recent surveillance of ‘most-at-risk population (MARPS) identified illicit substance use as a key behavior risks, with initiation to injecting drug use occurring as early as 11 years of age [33]. But the MARPs surveillance did not cover HCV (co)-infection, which significantly affects outcomes of HIV infection [34]. A situation analysis of HIV prevalence by key risk groups earlier revealed prevalence of HIV among IDU to be about 18% [35], while a separate study of IDU population in Western Kenya also put HIV prevalence in this demographic at comparable levels of 19% [17]. Although these prevalence data represented a significant rise of least 450% from low rates of 4% recorded years earlier [20], they are much lower than our reported HIV prevalence of 88% among the heroin injectors in Malindi. HCV is rarely included in most national surveillance studies, but independent report showed that 22% of IDUs in a cohort of about 300 injectors were HCV seropositive [36]. Other studies have reported much lower prevalence of HIV and HCV infections among injectors than we report in this paper [19, 37, 38]. Our study population may be highly biased towards HIV infection, particularly since the Omari rehabilitation facility is the only one known to offer both antiretroviral treatment and withdrawal management in Kenya. The facility may thus inherently attract more HIV infected injectors seeking multiple anonymous interventions and who are ready for behavior change. This may explain the high prevalence of HIV in this facility, which cannot be readily extrapolated to the general IDU community. Moreover, a number of IHUs had a past or active history of lawful confinement in facilities that lacked requisite sanitary and supportive systems to control transmission within those concentration settings. Together with generalized stigma associated with illicit drug use in Kenya, injectors tend to recede into closely neat communities of users, which we think promotes behavior patterns that facilitate sustained HIV transmission. The HIV prevalence data should therefore be treated contextually and with caution, as it may not reflect the general IHU/IDU population outside of rehabilitation settings. Consequently, the most important and informative infection data from our present cohort is that relating to HCV co-infection of the HIV infected heroin users.

Despite the paucity of evidence on co-infection rates, limited available data from IDU studies in Kenya show much higher infection rates than the general national population [39]. We show in this study that 18% of the HIV infected heroin injectors were co-infected with HCV and that 96% of all HCV cases were HIV-1 positive. This prevalence was within the range of the reported prevalence of 22% among a cohort of IDUs in Kenya [36]. By comparison, HCV infection was at least 3-fold less among HIV-1 negative injectors, with prevalence rates of 5.3%. These prevalence rates were higher for younger (40 years and below) and male than to older or female injectors. These subjects had no record of prior or current HCV-specific treatment. The existing national guideline for the management of infections among IDUs relies heavily on HIV treatment to mitigate infection burden [20]. Weaknesses exist in such protocol, in that HCV (and HBV)-mono infected individuals will not readily access treatment and care unless when screened for HIV, while co-infected IDUs do not receive optimal therapy for dual infection. A study of Canadian youths-at-risk found differences in gender-related risk factors that influenced initiation of IDU [40]. Although there were slightly more male than female IDUs in our study, the proportion of males that were mono or dual infected with HIV and HCV were higher than that of females. These differences could also likely be influenced by gender variability in healthcare-seeking behavior among Kenyans [41]. Importantly, the covertness of injection practice in Kenya, the stigma associated with HIV infection and the poor treatment coverage for both IDUs and HIV infected people are likely working in synergy to contribute to higher risk of infections in this group. For IDUs, this risk is exacerbated by poor and harmful injection practices such as sharing equipment [17]. This study did not look at hepatitis B virus (HBV) co-infection of either HIV or HCV, nor was similar data available for referencing from routine clinical procedures. Future investigations should benefit the field by generating such data.

HIV infection leads to adverse immunologic events due to CD4 T-cell depletion [42]. In people infected with HCV, co-infection with HIV compromises anti-HCV specific immunity while the dual infection environment induces adverse cellular and immune pathology [34, 43, 44]. Because of an increased risk of infection by these viruses among IDUs, we assessed the level of CD4 T-cells in blood and compared these between IDUs receiving anti-HIV medications and those naïve to ART, as well as between genders and infection status. Our data shows that IDUs receiving TDF or AZT regimens had significantly better CD4 T-cell levels than injectors receiving D4T while those receiving TDF also had better CD4 T-cell numbers than AZT recipients. We recently reported poorer virologic outcome in the D4T arm of a larger population of non-IDU patients on antiretroviral therapy [45]. The current data reinforce these earlier observations that D4T phase-out should be fast tracked at all levels of ART sequencing in to improve treatment outcome. Despite majority of the injectors being HIV mono-infected, dual infected IHUs had significantly depressed CD4 T-cell numbers than HIV mono-infected or uninfected injectors. HIV mono and HCV mono-infected injectors still had much lower CD4 T-cell counts than uninfected injectors. In deed, a Chi square model showed that IDUs had a significantly raised chance of being either dual or mono-infected with HIV/HCV. Our data is significant, as it demonstrates an increased risk of developing immune deficiency among IDUs who are behaviorally prone to multiple infections, particularly to HIV and HCV that share similar immune target cells. We did not prospectively follow up CD4 T-cell counts, but there is a wealth of data supporting their rapid decline among HIV-1 positive subjects, either among ART naïve individuals, or compared between injectors and non-injectors [42, 46, 47]. Our study offers useful information on the burden of mono and dual infections with the two viruses in IDU-concentration settings, and should be of significance in interpreting infection risks and designing mitigation strategies, while also alleviating adverse disease or treatment outcomes associated with multiple virus infections.

This study was limited to the extent that non-injectors were not included for comparison and ART status of some IDUs could not be verified. Nor could we ascertain how long the IDUs had been injecting heroin. These limitations are not unique to this study, as non-disclosure remains a pervasive cultural attribute in Kenya with reversal effect on treatment outcomes [48]. Finally, Malindi and Mombasa are contiguous coastal regions with immense international tourist activities. This, together with regional economic disparity, may contribute to bias in risk behaviors that could limit extrapolation of the findings to the general national population. Overall our data should be understood in the context of complexity of identifying populations that inject drugs in various settings, with majority of the available data lacking national demographic representation. Future studies should be designed whenever possible, with large nationally representative populations of both IDUs and non-injectors to improve data sensitivity.

## Conclusions

We have observed HIV-1 and HCV mono and dual infections among Kenyan IDUs from Malindi that was higher than demonstrated by any nationally available data. These infections were associated poor CD4 T-cell outcomes. To alleviate the increasing burden of infection in this group, we suggest fast-tracking integrated public health intervention approaches including (i) pre-exposure prophylaxis, (ii) harm-reduction including supply of individualized needles and syringes at care centers, (iii) substance replacement therapy, (iv) transitional aid from injecting to non-injecting use and (iv) infection screening and targeted test-and-treat ART for both HIV and HCV. Peer counseling should be considered to improve disclosure of drug use and uptake of preventive or therapeutic interventions.
